# Improvement in Red Soil Aggregate Stabilization Using Biopolymers

**DOI:** 10.3390/polym18212632

**Published:** 2026-10-23

**Authors:** Hai Lin, Qihang Lai, Yu Xia, Xiangyu Gong, Jinqi Zhu

**Affiliations:** 1 School of Infrastructure Engineering, Nanchang University, 999 Xuefu Avenue, Honggutan District, Nanchang 330031, China; linhai@ncu.edu.cn (H.L.);; 2 Engineering Research Center of Watershed Carbon Neutralization, Ministry of Education, Nanchang University, 999 Xuefu Avenue, Honggutan District, Nanchang 330031, China; 3 School of Resources & Environment, Nanchang University, 999 Xuefu Avenue, Honggutan New District, Nanchang 330031, China

**Keywords:** red soil, biopolymers, aggregate water stability, soil structure, stabilization mechanism

## Abstract

Red soils in southern China are highly vulnerable to water-induced structural breakdown and erosion because of their poor aggregate water stability. Although biopolymers have recently been proposed as environmentally friendly amendments for improving red soil stability, the links between their stabilization mechanisms and aggregate-level performance remain unclear. In this study, three representative biopolymers, namely calcium lignosulfonate (Ca-LS), xanthan gum (XG), and a bio-organic–mineral composite stabilizer (DK), were selected to improve the water stability of red soil aggregates. Controlled laboratory experiments were conducted to evaluate aggregate stability, microstructure, pore characteristics, and zeta potential of the amended soils. The results show that all three amendments improved aggregate water stability to different extents. XG showed the strongest overall stabilization within the tested conditions, whereas DK exhibited a gradual content-dependent improvement and Ca-LS displayed a non-monotonic response. Within the tested range, the optimal amendment contents for improving aggregate stability were 2.0% for DK, 1.5% for Ca-LS, and 1.0% for XG. In addition, biopolymer amendment increased the size of fragments generated after water-induced breakdown, suggesting a potentially lower susceptibility to detachment and transport. Microstructural analyses further showed that the amendments promoted particle cementation, network formation, and pore-structure adjustment, thereby enhancing aggregate stability through coupled interfacial bonding and structural reorganization. These findings provide a multiscale basis for understanding biopolymer-amended stabilization of red soil aggregates and for selecting suitable amendments for the remediation of degraded red soils.

## 1. Introduction

In the course of human economic and social development, the construction of various engineering facilities including water conservancy projects, dams, roads, railways and urban expansion has produced a large number of exposed engineering slopes and significantly changed the original geological conditions for rock and soil occurrence, resulting in increasingly serious soil and water loss in these areas. Under extreme conditions such as continuous heavy rainfall and rapid water level rise and fall, the erosion process is further aggravated [1,2]. Ecological restoration centered on vegetation recovery has been widely applied to control soil and water loss [3,4], while contributing to the reduction in regional carbon emissions. As the fundamental unit of soil structure, soil aggregates possess water stability that directly reflects their comprehensive capacity to resist rapid wetting, disintegration, dispersion and mechanical disturbance under rainfall or runoff conditions. Insufficient water stability of soil structure frequently causes massive loss of seeds, nutrients and topsoil, which hinders vegetation restoration from achieving the desired effects. Repeated replanting and remedial measures often incur additional economic costs and resource waste [5]. Therefore, the content and stability of soil water-stable aggregates are widely recognized as key indicators for evaluating soil erosion resistance [6].

Red soil is widely distributed in southern China and ranks among the most critical soil types for regional ecological restoration and land development. Nevertheless, it generally suffers from low organic matter content, weak aggregate stability and poor resistance to water-induced structural degradation, which renders it highly vulnerable to erosion [7]. Against the hot and humid climatic background of southern China, intense rainfall and concentrated seasonal runoff further accelerate the separation and migration of soil particles, which continuously exacerbates soil degradation and restricts regional sustainable development [8]. Soil structure acts as a core factor determining the functions of soil ecosystems, profoundly affecting hydrological processes, soil aeration, root development, microbial activity and organic matter turnover. A stable soil structure can provide favorable habitat conditions for the restoration of plant–soil systems [9,10,11,12]. As a typical special soil with poor water stability, red soil is highly susceptible to structural disintegration under the action of rainfall and surface runoff. Therefore, improving the water stability of red soil remains a key challenge for ecological restoration practices in humid subtropical regions.

The application of soil amendments to optimize soil structure and promote the formation and stabilization of soil aggregates has attracted increasing research attention. Traditional and nanomaterial stabilizers—such as cement, bentonite, micro-silica, and nano-TiO_2_ for loess stabilization [13] and Sarooj mortar combined with rice husk ash, lime, eggshell powder, polypropylene fibers, and nanosilica [14]—stabilize soil mainly through mineral cementation, pozzolanic reactions, and structural reinforcement, with their environmental compatibility and applicability closely tied to material composition and application conditions. By contrast, biopolymer amendments act through adsorption, particle bridging, molecular-chain interactions, and interfacial bonding. Additionally, aggregate stability is generally closely correlated with soil organic matter accumulation and microbial activity. Biopolymer materials can contribute substantially to the short-term formation and stabilization of soil aggregates in various restoration practices through mechanisms including particle bridging, electrostatic regulation and interparticle bonding. However, the mechanisms and quantitative effects of different biopolymer materials on red soil improvement still require systematic and in-depth exploration.

Existing studies have demonstrated that different types of biopolymer materials affect soil aggregate structure mainly through three pathways: physical bonding, chemical bridging and biological cementation [15]. Anionic polyacrylamide (PAM) is one of the most representative polymeric soil amendments. It can adsorb onto mineral surfaces and form polymer bridges between soil particles, thereby improving the water stability of soil aggregates and resistance to dispersion [16,17]. This effect is particularly prominent in sandy soils or soils with low organic matter content [16,18]. Biochar possesses both physical supporting effects and surface chemical reactivity. It not only provides rough surfaces and pore spaces for the attachment of fungal hyphae and colloids, but also regulates soil moisture conditions and ion exchange environments, thereby enhancing aggregate stability [19,20]. Organic amendments including compost, farmyard manure and partial fertilization treatments promote the generation of microaggregates and macroaggregates by increasing soil organic carbon content, facilitating the formation of clay–organic complexes and stimulating microbial activities [21]. In recent years, microbial inoculation and indigenous microbial community stimulation technologies have also gained increasing attention. They can boost the production of extracellular polymeric substances including polysaccharide and protein colloids, cement fine particles at the microscopic scale and facilitate the formation of micro-aggregates [22]. Notably, unlike synthetic polymers, biochar and traditional organic amendments, which primarily exert direct physical, chemical or long-term organic improvement effects, biopolymer materials tend to trigger changes in aggregate structure relatively rapidly through immediate bridging, bonding and interfacial regulation. However, their dose-dependent responses, interactions with soil texture and ionic composition, as well as environmental adaptability, have not been fully characterized by clear quantitative indicators. This limitation restricts the development of reliable, parameterized guidelines for field application and hinders the effective assessment of the materials’ potential environmental risks and applicability across different soil types.

Biopolymers are generally rich in surface-active functional groups such as carboxyl, hydroxyl, amino and sulfonate groups, and feature tunable molecular chain configurations and surface charge properties. These properties allow many bio-based amendments to regulate interfacial interactions through mechanisms including electrostatic adsorption, ionic bridging, molecular-chain entanglement, and surface adhesion [23,24,25]. Different from amendments that mainly rely on organic matter accumulation or mineral cementation, biopolymer materials directly mediate soil structural reorganization at the initial stage of aggregate formation. This mechanism is distinctly different from the gradual and indirect action patterns of organic and mineral amendments.

Previous studies have largely examined individual biopolymers or evaluated their macroscopic stabilization performance, whereas comparative analyses linking amendment chemistry with aggregate-scale responses remain limited. In particular, the links between stabilization mechanisms and aggregate-level performance remain insufficiently understood in red soil. Highly weathered and rich in iron and aluminum oxides, red soil exhibits complex surface charge characteristics and high mineral reactivity. The above characteristics may endow bio-based biopolymers with unique and yet insufficiently revealed action mechanisms in red soil. Against this background, three environmentally friendly biopolymeric or bio-organic amendments, namely xanthan gum (XG), calcium lignosulfonate (Ca-LS), and a bio-organic–mineral composite stabilizer (named DK), were selected as red soil amendments in this study. Through the indicators of particle size distribution, mean weight diameter, water-stable aggregate content and the water stability of soil aggregates, the improvement effects of these biopolymers on red soil were quantitatively evaluated. By combining aggregate-stability measurements with SEM, FTIR, pore-structure, and zeta-potential observations, this study compared the responses of the three amendments and proposed plausible interfacial and structural processes associated with their stabilizing effects. The research findings are expected to provide multiscale scientific support for the screening of environmentally friendly amendments and the formulation of ecological restoration strategies.

## 2. Materials and Methods

### 2.1. Experimental Materials

**Figure 1:**
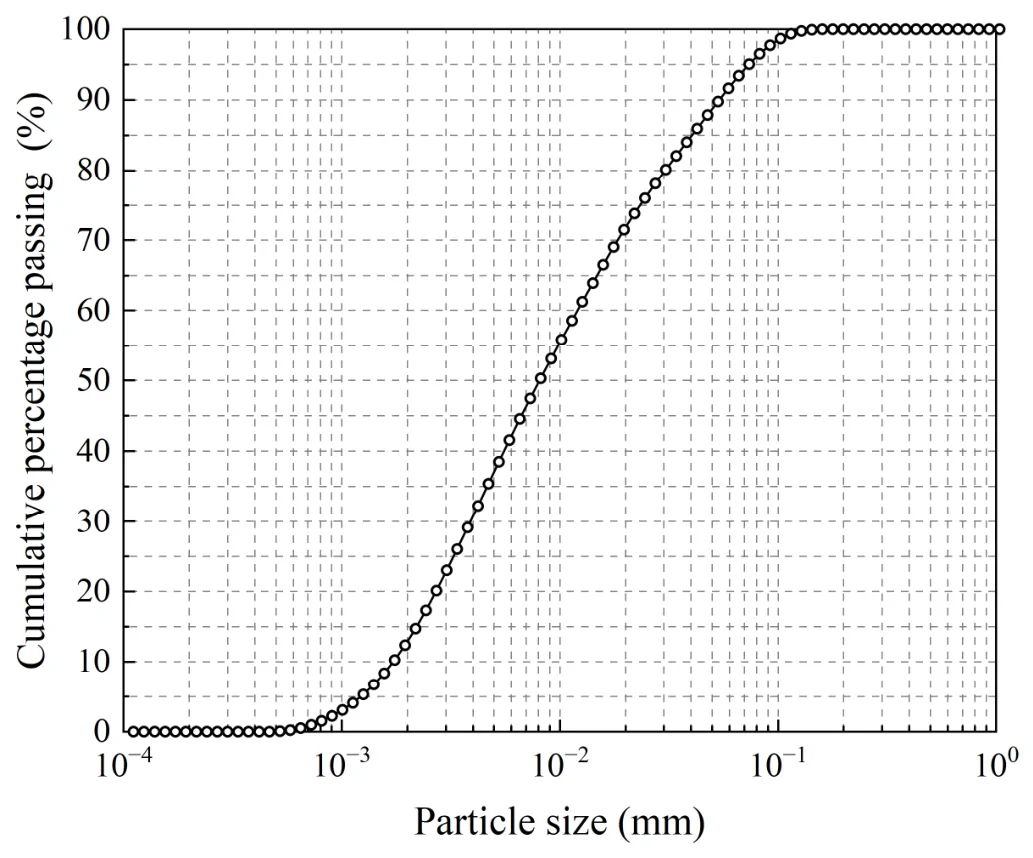
Particle size distribution curve of the tested red soil.

The red soil used in this study was collected from Nanchang, Jiangxi Province, China. After removing visible plant roots, gravel, and other impurities, the soil was air-dried and passed through a 2 mm standard sieve for subsequent sample preparation. The naturally air-dried water content of the soil was 3.6%, and the specific gravity of soil solids was 2.76. The liquid limit (LL), plastic limit (PL), and plasticity index (PI) were 40.7%, 18.9%, and 21.8%, respectively. A standard Proctor compaction test was conducted in accordance with GB/T 50123-2019 [26] to characterize the compaction properties of the untreated red soil, yielding a maximum dry density of 1.80 g/cm^3^ and an optimum water content of 16.1%. The particle size distribution was 4.7% sand (d > 0.075 mm), 58.5% silt (0.005 mm < d < 0.075 mm), and 36.8% clay (d < 0.005 mm) (Figure 1).

**Figure 2:**
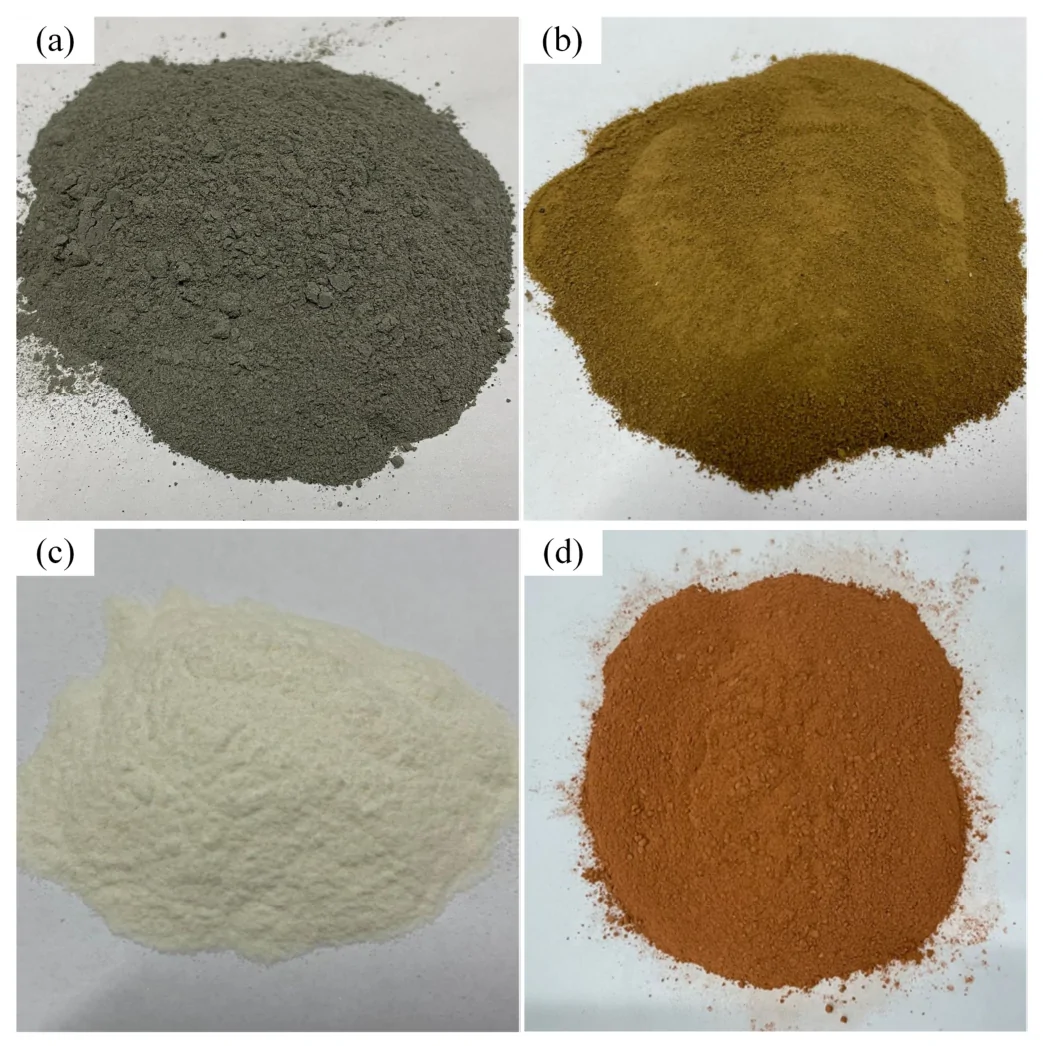
Three biopolymer soil amendments used in this study: (**a**) DK; (**b**) Ca-LS; (**c**) XG; (**d**) CK.

DK is a bio-organic–mineral composite stabilizer supplied by Nanjing Yichen Environment Technology Co., Ltd. (Nanjing, China). It appears as a dark gray powder and is readily dispersible in water at ambient temperature (Figure 2a). Composition analysis indicated that DK consisted mainly of inorganic minerals, accounting for approximately 76.9% of its mass, while the remaining 23.1% comprised bio-based organic components rich in polypeptide- and amino-acid-like substances derived from microbial metabolic products. Calcium lignosulfonate (Ca-LS), an industrial-grade lignin derivative, appears as a brownish-yellow powdery solid (Figure 2b) with a faint aromatic odor. It is highly soluble in water at ambient temperature, with a pH value ranging from 6 to 8 and a molecular weight of 1800–2500 Da. Its composition consists of 93–97% dry matter and 3–7% moisture, among which calcium lignosulfonate accounts for 50–55% and reducing substances account for 4–8%. Ca-LS exhibits excellent stability, dispersibility, adhesiveness, and chelating capacity, and is a non-toxic, non-corrosive, and environmentally friendly anionic surfactant based on biopolymer. Xanthan gum (XG) used in this study was of food grade. It appears as a pale yellow powdery solid (Figure 2c) with a slight odor and is readily soluble in water. The XG had a loss on drying of 7.3% and an ash content of 10.6%. XG is a polysaccharide-based biopolymer compound produced by the fermentation of corn starch using Xanthomonas campestris. It is non-toxic, neutral, environmentally friendly, and renewable, existing as a polyanion in aqueous solutions, thus classified as an important biopolymer. This material exhibits excellent advantages including high viscosity, high temperature resistance, acid–alkali resistance, and salt stability, and is commonly used as a stabilizer, thickener, and suspending agent. The three amendments were evaluated in their commercially available forms to better represent their direct use in practical soil improvement and ecological restoration. Accordingly, the amendment contents reported in this study were based on the masses of the commercial amendment products. For comparison, the unamended red soil (CK) is shown in Figure 2d.

### 2.2. Specimen Preparation

Each commercial amendment product was added at four non-zero contents (0.5%, 1.0%, 1.5%, and 2.0% by dry soil mass), together with an unamended control (Table 1). Initially, the soil and amendments were mixed thoroughly in a dry state at a low stirring speed. Subsequently, the stirring speed was adjusted to the maximum setting, while atomized deionized water was sprayed into the mixture using a sprayer, with continuous rapid stirring during water addition. The volume of water added was calculated based on the difference between the target initial water content (18%) of the final soil specimens and the water content of the air-dried soil. The target initial water content of 18% was selected based on the representative field moisture condition of the red soil to better approximate its in situ moisture state. After the required amount of water had been added, the mixer was stopped, and soil adhering to the bottom and inner wall of the mixing bowl was manually scraped off and remixed with the bulk soil. The mixture was then mechanically stirred at the maximum speed for approximately 10 s, followed by one additional cycle of manual remixing and mechanical stirring for approximately 5 s. Soil aggregates formed spontaneously during the mechanical mixing and wetting process, and no artificial molding or compaction was applied during aggregate preparation. The same specimen-preparation procedure was applied to all amended treatments and the unamended control. Following thorough mixing, the soil mixture was sealed and allowed to stand for 24 h to ensure uniform water distribution throughout the soil matrix. Finally, amended red soil specimens with different biopolymer types and amendment contents were obtained. Based on the amendment type, the amended red soils were abbreviated as DK-red soil, Ca-LS-red soil, and XG-red soil, respectively. The red soil without any amendments served as the control group (CK), referred to as unamended red soil.

### 2.3. Experimental Methods

#### 2.3.1. Soil Aggregate Stability Assay

Water stability of soil aggregates was determined using the Le Bissonnais (LB) method [27], which is incorporated into the international standard ISO 10930:2012 [28]. The detailed procedures were as follows. All red soil samples were first dry-sieved through 3 mm and 5 mm standard sieves. The fraction passing the 5 mm sieve but retained on the 3 mm sieve was collected as the 3–5 mm aggregate fraction and dried in a forced-air drying oven at 40 °C for 24 h to achieve constant mass, after which three distinct treatments were conducted: (1) Fast wetting (FW): 5–10 g of 3–5 mm soil aggregates were weighed and gently immersed in 50 mL of deionized water in a 250 mL beaker. After standing for 10 min, excess water was removed with a pipette, and the aggregates were rinsed into a 0.05 mm copper sieve submerged in ethanol using an ethanol wash bottle. (2) Slow wetting (SW): 5–10 g of 3–5 mm soil aggregates were placed on filter paper, which was then placed in a 250 mL beaker lined with a 3 cm thick sponge. Deionized water was added to reach the 2.5 cm mark on the sponge. After standing for 60 min, the aggregates were rinsed into a 0.05 mm copper sieve submerged in ethanol. (3) Wet stirring (WS), representing mechanical breakdown after pre-wetting: 5–10 g of 3–5 mm soil aggregates was gently immersed in 50 mL of ethanol in a 250 mL beaker. After 30 min of standing, excess liquid was removed with a pipette. The aggregates were then flushed into an Erlenmeyer flask containing 50 mL of deionized water, and additional deionized water was added along the inner wall of the flask to bring the total volume to 200 mL. The flask was sealed with a stopper, inverted, and shaken 10 times. After standing for 30 min, excess water was removed with a pipette, and the aggregates were rinsed into a 0.05 mm copper sieve submerged in ethanol. Following all three treatments, the same subsequent procedures were performed: The 0.05 mm copper sieve was spirally agitated five times in ethanol with uniform force. Aggregates retained on the sieve were rinsed into an evaporating dish and dried in a forced-air drying oven at 40 °C for 48 h. Finally, the dried aggregates were passed through a nest of sieves (2, 1, 0.5, 0.2, 0.1, and 0.05 mm), and the mass of aggregates in each size class was measured to the nearest 0.0001 g to calculate their mass fractions.

The test indicator for soil aggregate stability, the mean weight diameter (MWD), is calculated using the following formula:

(1) $$\mathrm{MWD}=\sum_{i=1}^{n}x_{i}w_{i}$$

*x_i_* is the mean diameter of aggregates in the *i*-th particle size fraction (mm), *i* = 1, 2, 3, 4, 5, 6, 7; *w_i_* is the mass percentage of aggregates in the *i*-th particle size fraction relative to the total aggregate mass (%). Based on the classification proposed by Le Bissonnais (1996) [27], aggregate stability was categorized according to MWD as follows: very unstable, MWD < 0.4 mm; unstable, 0.4 ≤ MWD < 0.8 mm; medium, 0.8 ≤ MWD < 1.3 mm; stable, 1.3 ≤ MWD ≤ 2.0 mm; and very stable, MWD > 2.0 mm.

#### 2.3.2. Scanning Electron Microscopy (SEM) Test

To investigate the soil particle bonding and pore-filling characteristics in the microstructure of biopolymer unamended and amended red soils, a TESCAN MIRA LMS scanning electron microscope (SEM) was used for imaging and observation. Aggregate samples with a particle size of 3–10 mm were collected from both unamended and amended red soils, oven-dried at 60 °C, and then subjected to SEM imaging analysis. The total number of test samples was 4. For SEM observation, representative subsamples were composited from each treatment. Multiple fields of view were examined for each specimen, and representative images were selected for microstructural interpretation. SEM images were used for qualitative microstructural evidence rather than statistical comparison.

#### 2.3.3. Nitrogen Adsorption–Desorption Test

To quantitatively evaluate the specific surface area, average pore diameter, and pore size distribution characteristics of unamended and amended red soils, a BSD-PS2 specific surface area and pore size analyzer, which is manufactured by Best Instrument Technology (Beijing, China) Co., Ltd. based in Beijing, was employed. Aggregates with a particle size of 3–5 mm were collected from both unamended and amended red soils and dried in a forced-air drying oven at 40 °C for 24 h. An electronic balance with a precision of 0.0001 g was used to weigh 0.7–1.0 g of the oven-dried soil aggregate specimens for the nitrogen adsorption–desorption test. The specimens were tested at 77.3 K using N_2_ (nitrogen) as the adsorbate. The specific surface area was calculated using the BET method, whereas the pore-size distribution was obtained using the BJH method implemented in the instrument software. Because the BJH method has limited reliability for quantitative characterization of pores below approximately 2 nm, the <2 nm region was excluded from quantitative interpretation [29]. The total number of test samples was 26.

#### 2.3.4. Fourier Transform Infrared (FTIR) Spectroscopy Test

The principal functional groups of DK, Ca-LS, and XG were characterized by Fourier transform infrared (FTIR) spectroscopy using an FTIR-850 spectrometer (Tianjin, China). Representative samples of the three biopolymeric amendments were ground and passed through a 100-mesh sieve with an aperture of 0.15 mm. The sieved powder particles were then oven-dried at 105 °C for 10 h to ensure complete moisture removal from the specimens. A small amount of the dried powder was mixed uniformly with potassium bromide (KBr) in a specific ratio and pressed into thin pellets under static pressure, which were then placed into the spectrometer for FTIR testing. In addition, unamended red soil and red soils amended with 1% and 2% DK, 1% and 2% Ca-LS, and 1% and 2% XG were subjected to FTIR analysis to evaluate amendment-soil interfacial interactions.

Peak positions in the soil FTIR spectra were identified using a consistent peak-search procedure in OriginPro 2024 with the Min & Max baseline mode, negative peak direction, a local-maximum search method, 15 local points, and a height threshold of 10%.

#### 2.3.5. Zeta Potential Analysis

To investigate the surface charge characteristics of colloidal particles in unamended red soil and amended red soils, a 90Plus PALS zeta potential analyzer, which is manufactured by Brookhaven Instruments Corporation (Holtsville, NY, USA), was employed. Representative aggregates from both unamended and amended red soils were collected, dried in a forced-air drying oven at 40 °C for 24 h, ground, and sieved through a 200-mesh sieve (aperture size: 0.074 mm). For zeta-potential measurements, the sieved soil was dispersed in deionized water, which served as the measurement medium, and magnetically stirred for 15 min to promote uniform dispersion and minimize particle aggregation. No background electrolyte was added, and the suspension pH was not externally adjusted. For suspensions containing relatively coarse particles, the particles were allowed to settle and the supernatant was collected for measurement. The solid-to-liquid ratio was adjusted according to the instrument count rate, which was maintained at no less than 500 kcps. Freshly prepared or diluted suspensions were allowed to equilibrate for 5–20 min before analysis. All treatments were prepared and measured following the same procedure and measurement criteria to ensure comparability among treatments. A total of 13 treatment combinations were tested, with two samples analyzed for each treatment, resulting in 26 samples. The experimental design for the red soil improvement mechanism is shown in Table 2.

## 3. Results

### 3.1. FTIR Characteristics of the Amendments and Amended Red Soils

**Figure 3:**
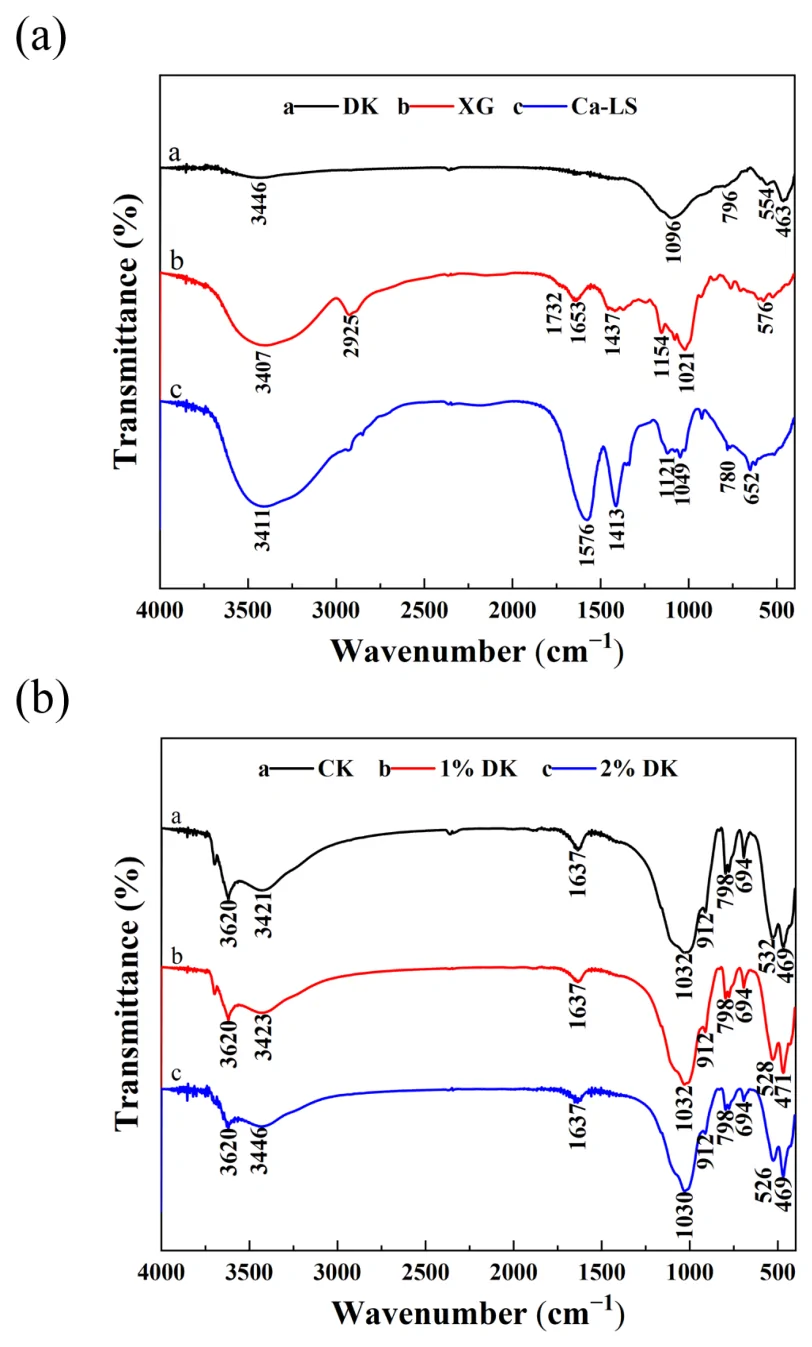

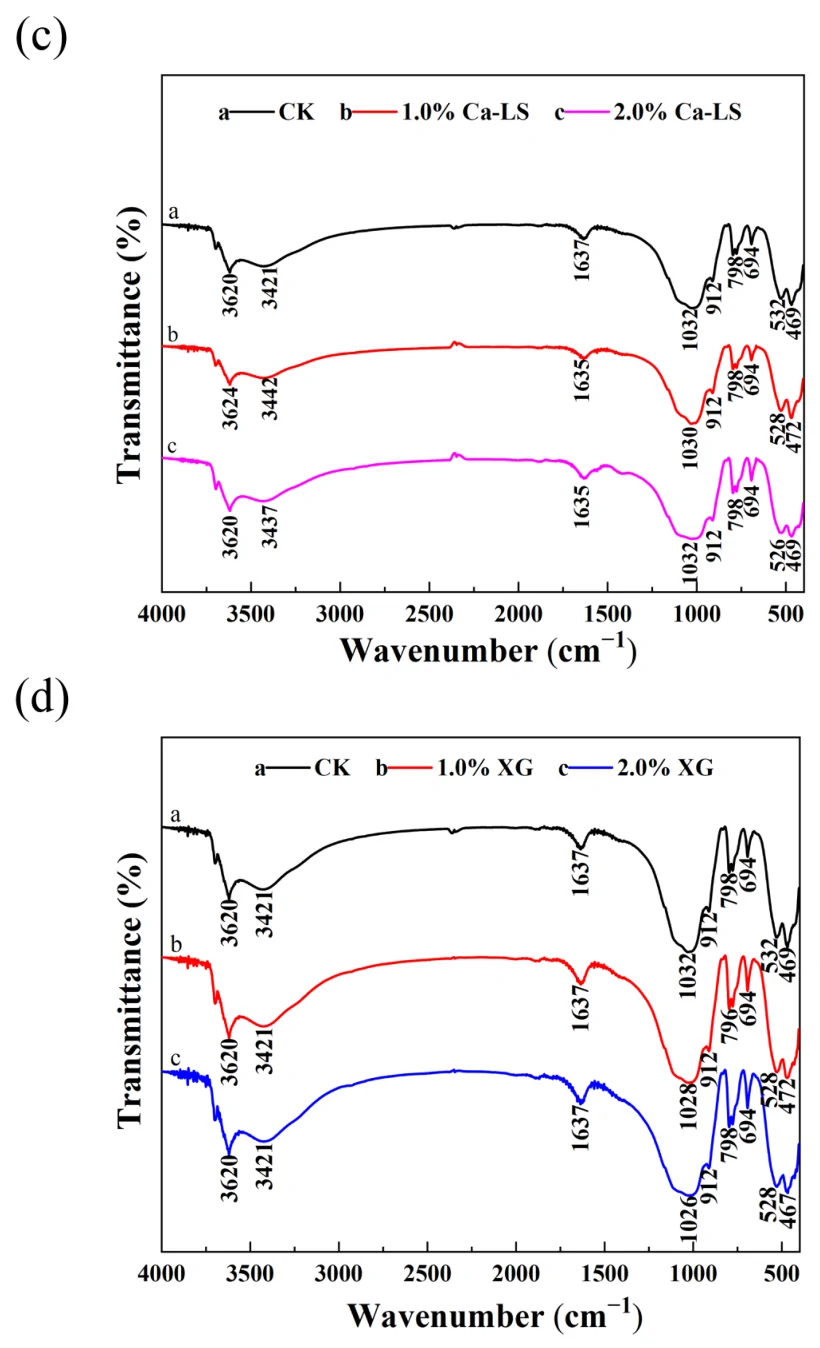
FTIR spectra of (**a**) DK, Ca-LS, and XG amendments; (**b**) unamended and DK-amended red soils; (**c**) unamended and Ca-LS-amended red soils; and (**d**) unamended and XG-amended red soils.

The FTIR spectrum of DK exhibited characteristic absorption peaks at 3446 cm^−1^ (O–H/N–H) and 1096, 796, and 463 cm^−1^ (Si–O stretching and bending), confirming the coexistence of organic components bearing hydroxyl/amino groups and an inorganic mineral phase. The spectrum of xanthan gum showed characteristic absorption peaks at 3407 cm^−1^ (–OH), 1732 cm^−1^ (carbonyl C=O), 1653 cm^−1^ (–COO^−^), and 1154/1021 cm^−1^ (glycosidic C–O–C/C–O), confirming its polysaccharide structure bearing hydroxyl, acetyl, and glucuronic-acid (carboxylate) groups. For calcium lignosulfonate, characteristic absorption peaks appeared at 3411 cm^−1^ (–OH), 1576/1413 cm^−1^ (aromatic C=C), and 1121/1049 cm^−1^ (sulfonate S=O), confirming its hydroxyl, aromatic, and sulfonate functionalities (Figure 3a). In summary, the three amendments differed in their dominant functional groups: DK was characterized by hydroxyl/amino and Si–O groups (an organic–mineral composite), xanthan gum by carboxylate and glycosidic groups (an anionic polysaccharide), and calcium lignosulfonate by aromatic and sulfonate groups (a lignin derivative).

Comparison of the FTIR spectra of unamended and amended red soils revealed treatment-dependent changes in several absorption bands (Figure 3b–d). For DK-amended red soil, the broad O–H stretching band near 3421 cm^−1^ shifted slightly to approximately 3423 cm^−1^ at 1% DK and exhibited a more pronounced blue shift to approximately 3446 cm^−1^ at 2% DK. For Ca-LS-amended red soil, the O–H stretching band exhibited a blue shift from approximately 3421 cm^−1^ to 3442 and 3437 cm^−1^ at Ca-LS contents of 1% and 2%, respectively. For XG-amended red soil, the O–H stretching band remained near 3421 cm^−1^, whereas the band near 1032 cm^−1^ shifted to lower wavenumbers, reaching approximately 1028 and 1026 cm^−1^ at XG contents of 1% and 2%, respectively. These treatment-dependent spectral changes indicate alterations in the local bonding environment after amendment addition and provide spectroscopic evidence consistent with amendment–soil interfacial interactions.

### 3.2. Microstructural Characteristics

**Figure 4:**
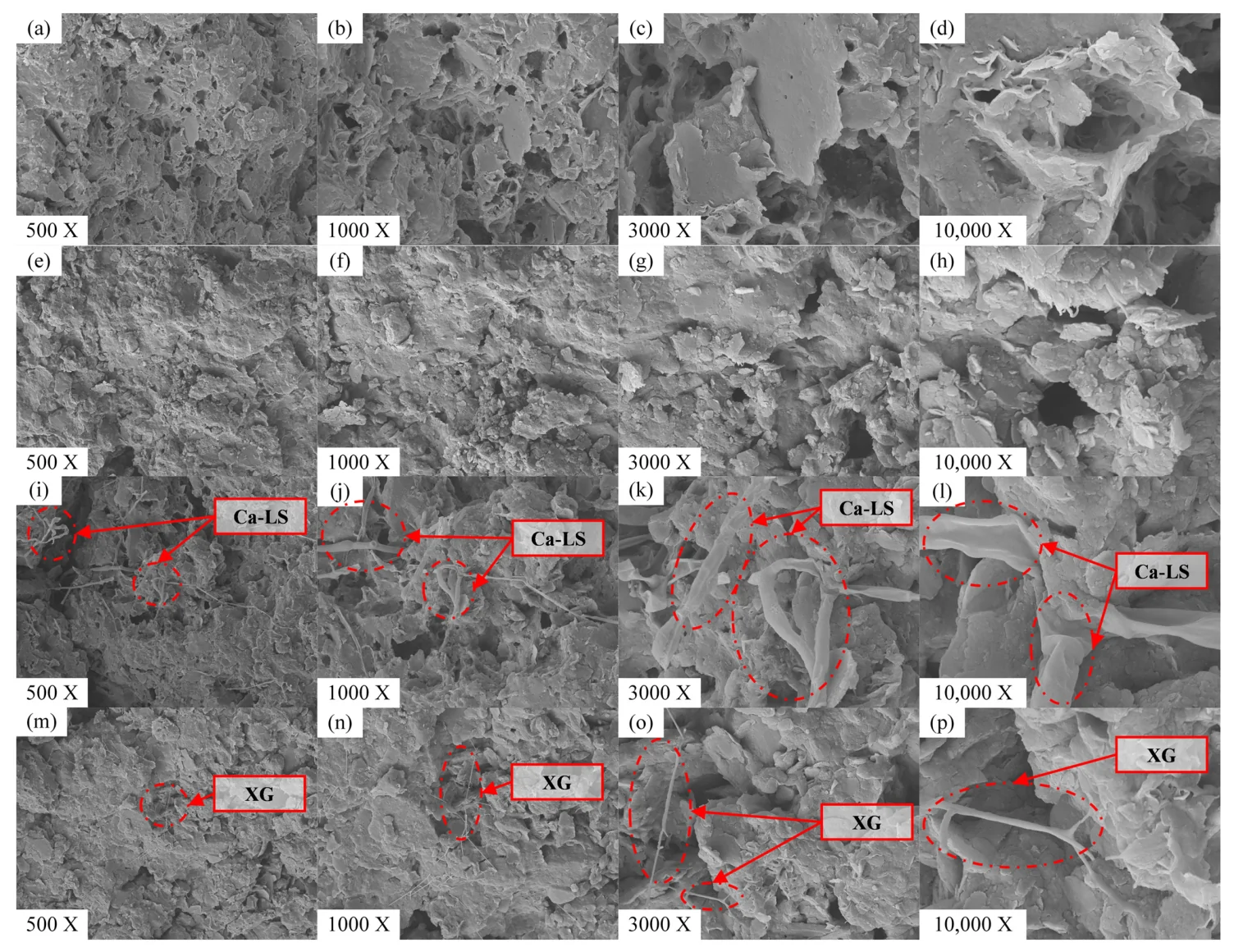
SEM images of aggregate surfaces of red soil: (**a**–**d**) unamended red soil; (**e**–**h**) 1.5% DK; (**i**–**l**) 1.5% Ca-LS; (**m**–**p**) 1.5% XG.

According to the analysis of scanning electron microscopy (SEM) images (Figure 4), the unamended red soil exhibited typical microstructural characteristics of a low-plasticity clay, with distinct soil particle boundaries, sharp edges and corners, weak interparticle connections, extensive pore distribution, and a loose overall structure. After the incorporation of soil amendments, the microstructures of the amended red soils became denser than that of the unamended red soil, although the morphological characteristics differed among amendments. The DK-amended soil (e–h) mainly exhibited a denser microstructure with fewer visible pores and enhanced interparticle contact, whereas Ca-LS (i–l) and XG-amended soils (m–p) showed more recognizable cementing structures associated with particle connection. In Ca-LS- and XG-amended soils, these cementing substances adhered to particle surfaces, connected adjacent soil particles, and partially filled interparticle pores. Particularly in XG-amended soil, the filamentous cementing substances contributed to the formation of a three-dimensional (3D) spatial network within the soil microstructure, enhancing particle connections. This 3D network structure restricts the displacement of soil particles under external forces, exerts a certain reinforcing effect on the soil, binds the soil particles tightly together, and thus enhances the firmness and stability of the soil structure. While filling some of the original small pores, the cementing substances also form new, larger pores between aggregates, thereby optimizing the pore distribution. Notably, the thickness of the filamentous cementing substances in the microstructures of the three amended red soils differed: The morphology of cementing substances varied among amendments. Ca-LS exhibited more evident connecting features between soil particles, XG showed filamentous cementing structures associated with a 3D network, and DK mainly showed structural densification with reduced visible pores.

### 3.3. Specific Surface Area and Pore Characteristics

**Figure 5:**
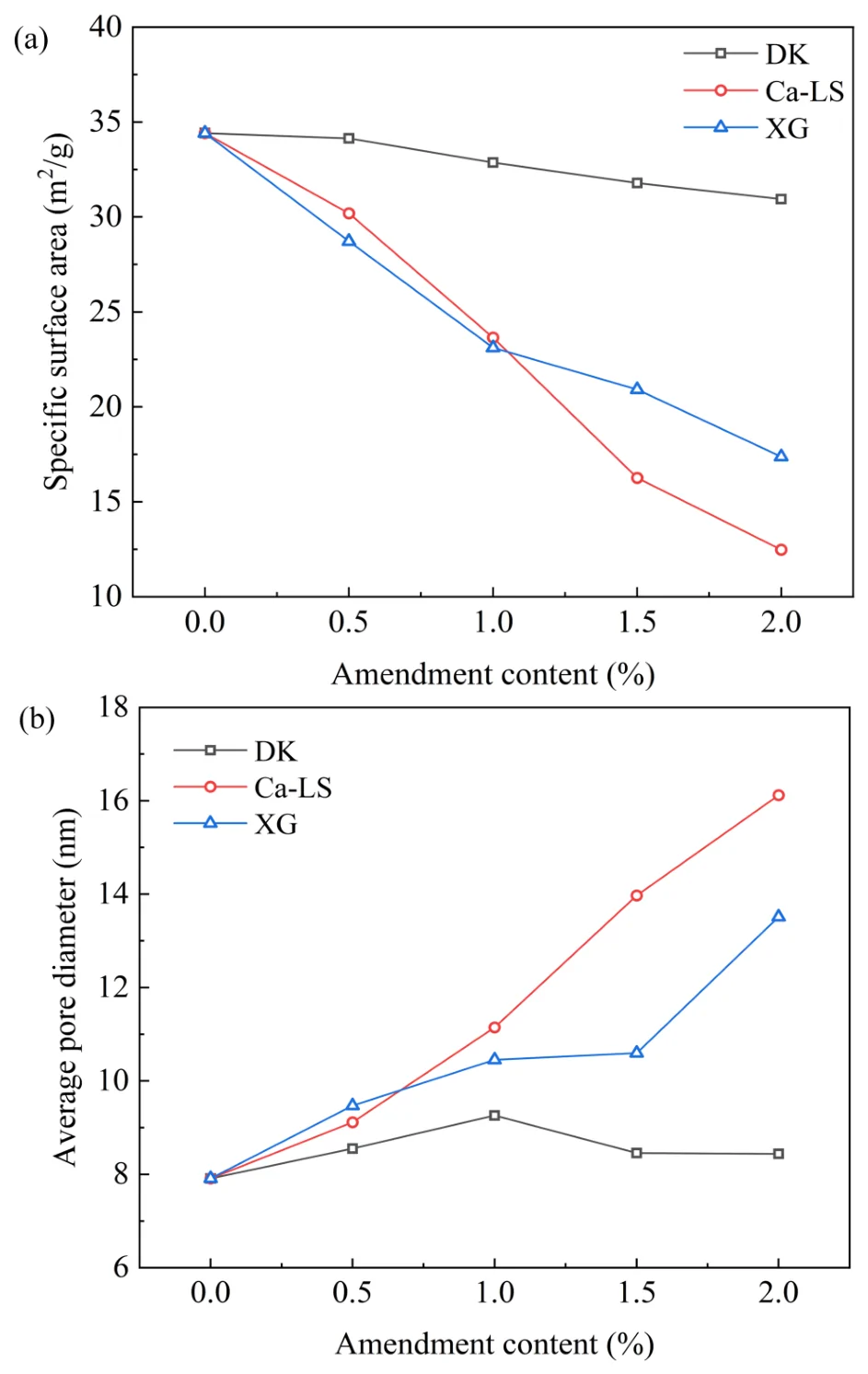
Specific surface area and average pore diameter of amended red soil: (**a**) specific surface area; (**b**) average pore diameter.

Specific surface area (SSA) and average pore diameter (APD) are critical parameters for quantifying soil surface properties and pore structure. As shown in Figure 5, the SSA and APD of unamended red soil were 34.42 m^2^/g and 7.91 nm, respectively. Upon individual application of the three different amendments, the SSA of red soil consistently decreased with increasing amendment content, while the APD within red soil aggregates increased to varying degrees. Within the applied amendment content range, the SSA of DK-amended, Ca-LS-amended, and XG-amended red soils could be reduced to a minimum of 30.94, 12.48, and 17.37 m^2^/g, respectively, representing reductions of 10.11%, 63.74%, and 49.54% compared with the unamended red soil. In contrast, the APD of DK-amended, Ca-LS-amended, and XG-amended red soils could be increased to a maximum of 9.26, 16.12, and 13.51 nm, respectively, corresponding to increases of 17.07%, 103.79%, and 70.80% relative to the unamended red soil.

**Figure 6:**
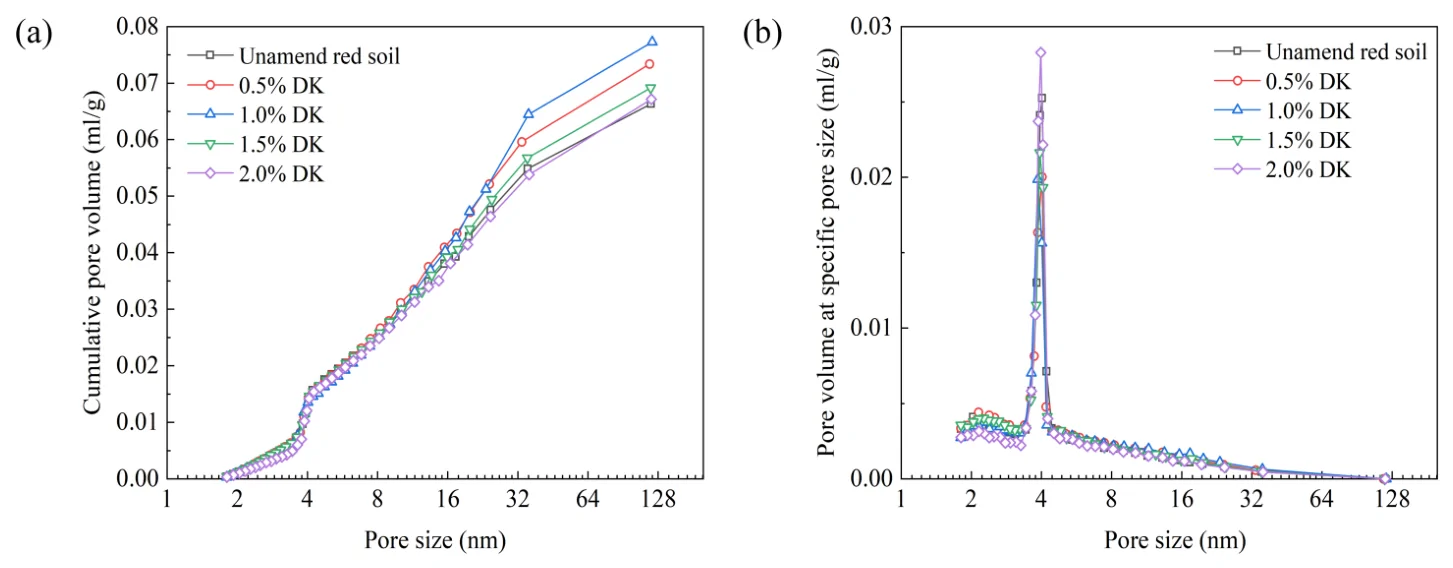

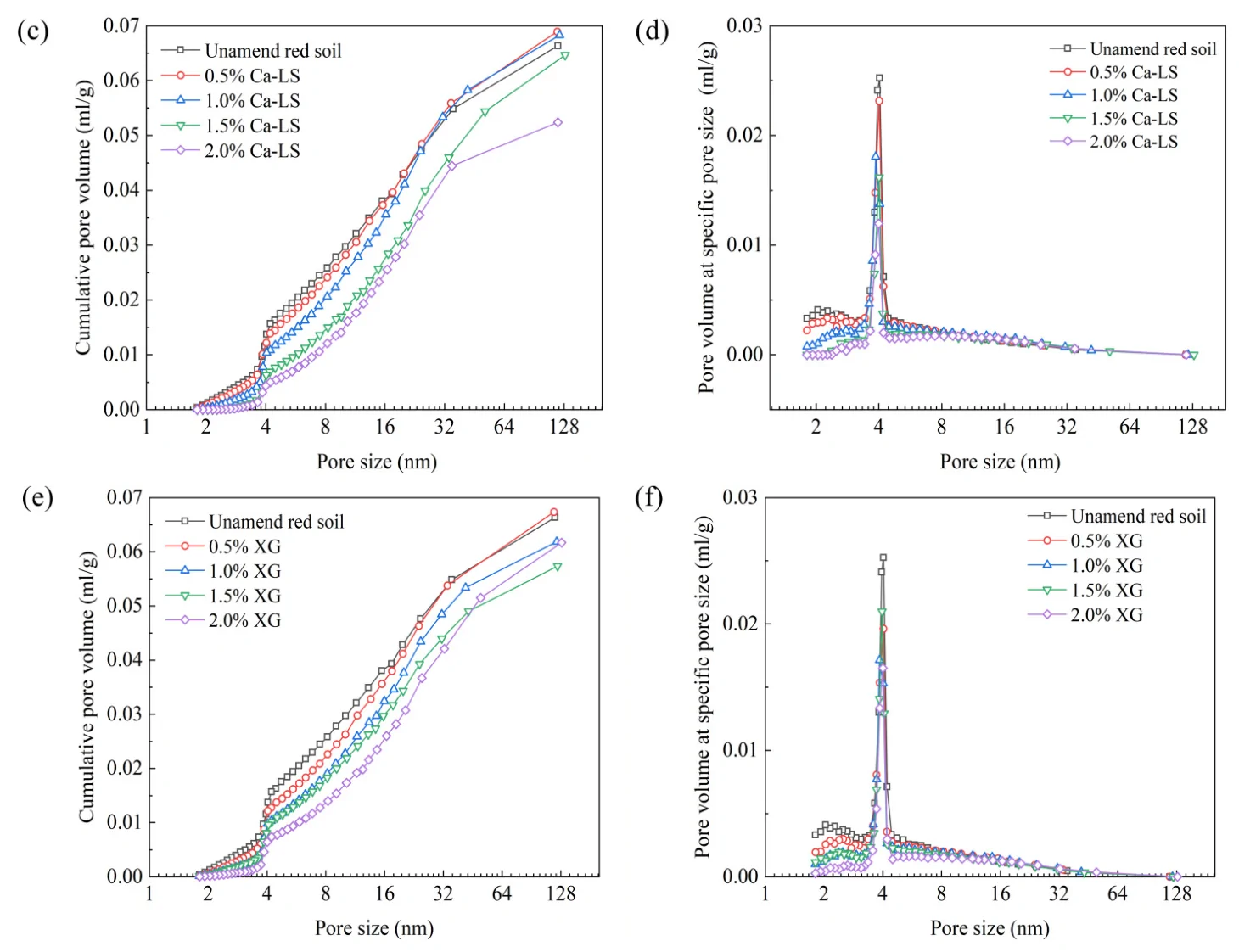
Cumulative pore volume and pore size distribution curves of red soil under different treatments and different amendment contents: (**a**,**b**) DK; (**c**,**d**); Ca-LS; (**e**,**f**) XG.

For quantitative interpretation of the BJH-derived pore-size distribution, pores ≥ 2 nm were considered in four size fractions: 2–5 nm, 5–10 nm, 10–50 nm, and >50 nm (Figure 6). The <2 nm region was not quantitatively interpreted because of the limited applicability of the BJH method in the micropore range. The latter half of the cumulative pore volume curve of DK-amended red soil was generally shifted upward compared with that of the unamended red soil. The total pore volume of DK-amended red soil exhibited a trend of first increasing and then decreasing with increasing amendment content, but it was consistently higher than that of the unamended red soil. For the unamended red soil, the proportions of pore volume in the 2–5 nm, 5–10 nm, 10–50 nm, and >50 nm fractions relative to the total pore volume were 25.93%, 16.95%, 37.92%, and 17.32%, respectively. The addition of DK, Ca-LS, and XG altered the pore size distribution characteristics of red soil aggregates. With the increase in amendment content, the left half of the pore size distribution curve shifted downward, while the right half shifted slightly upward, which was characterized by changes in the 2–10 nm pore fractions and an increase in the proportion of pores in the 10–50 nm range.

Specifically, following DK amendment, the proportions of pores in the 2–5 nm, and 5–10 nm ranges decreased to 25.03%, and 16.52%, corresponding to reductions of 3.47%, and 2.54%, respectively. By contrast, the proportion of pores in the 10–50 nm range increased by 18.7% to 45.01%. DK amendment therefore shifted the BJH-resolved mesopore distribution toward relatively larger pore sizes to a certain degree. A more pronounced effect was observed under Ca-LS treatment: with increasing amendment content, total pore volume in red soil declined, and the proportion of 2–5 nm pores fell to 12.27% (a 52.68% reduction). The proportions of 5–10 nm and 10–50 nm pores increased to 18.47% and 54.07%, representing increments of 8.97% and 42.59%, respectively. Ca-LS treatment therefore markedly altered the mesopore-size distribution, particularly by reducing the 2–5 nm fraction and increasing the 10–50 nm fraction. XG treatment induced a similar trend in pore structure evolution. As the amendment content increased, the proportions of 2–5 nm and 5–10 nm pores decreased to 13.98% and 13.9%, with reductions of 46.09%, and 17.99%, respectively, whereas the proportion of 10–50 nm pores rose markedly to a maximum of 55.35%, an increase of 45.97%. Overall, the amendments altered the BJH-resolved pore-size distribution toward relatively larger pore sizes, particularly within the 10–50 nm range. Such an improved distribution might be enhanced pore connectivity and air–water conductivity, establishing a more favorable physical environment for plant root growth, microbial activity, and nutrient migration.

### 3.4. Aggregate Stability Characteristics of Amended Red Soil

**Figure 7:**
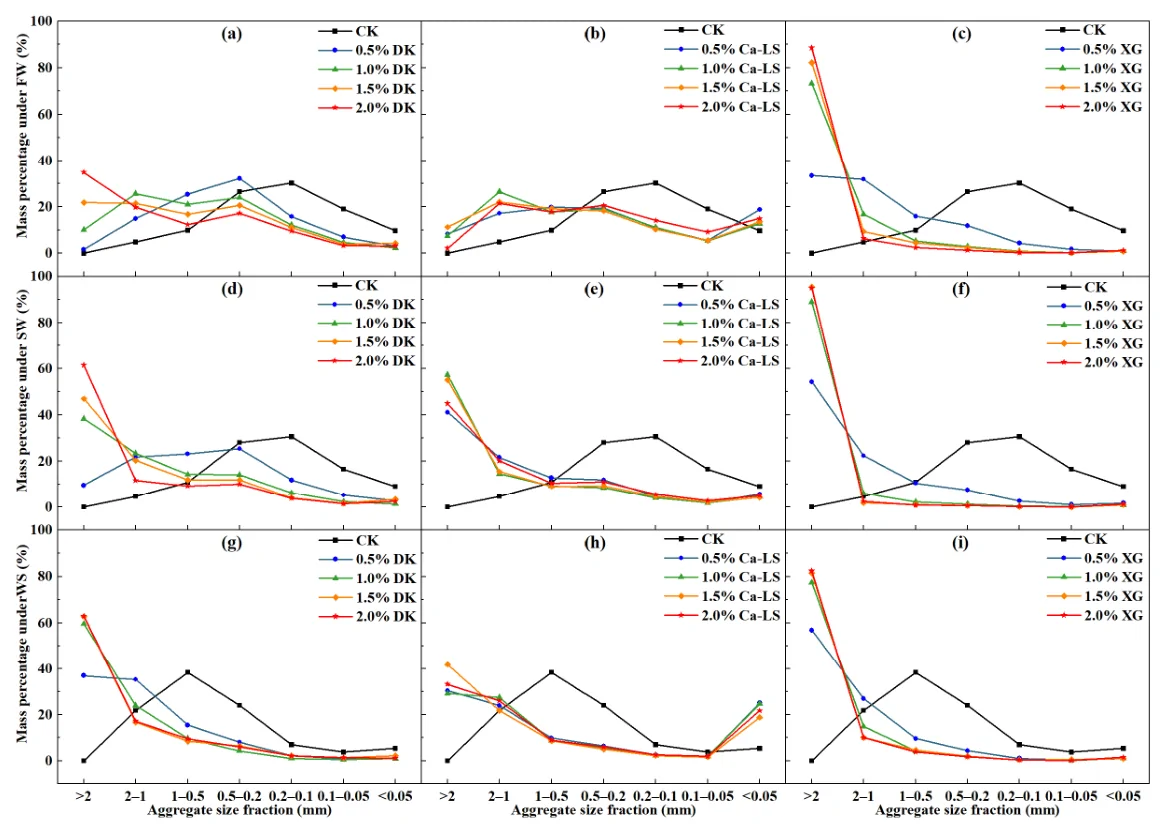
Aggregate-size distribution profiles of unamended and amended red soils under different Le Bissonnais treatments. Panels (**a**–**c**), (**d**–**f**), and (**g**–**i**) correspond to FW, SW, and WS, respectively, for DK-, Ca-LS-, and XG-amended red soils.

**Figure 8:**
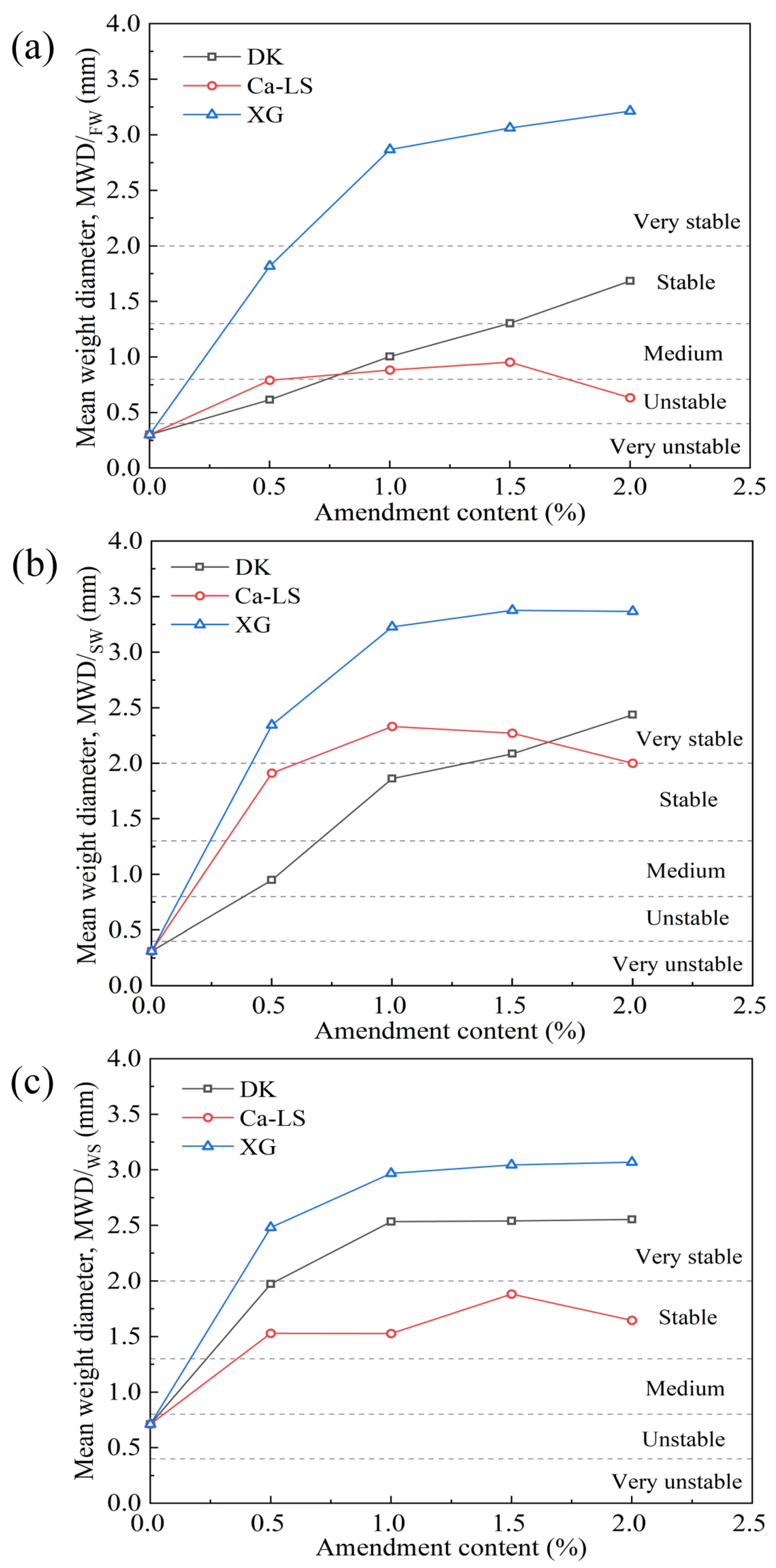
Aggregate stability characteristics of amended red soil: (**a**) fast-wetting treatment; (**b**) slow-wetting treatment; (**c**) wet-stirring treatment.

The aggregate-size distribution profiles of amended red soil under FW, SW, and WS treatments are shown in Figure 7, whereas the corresponding aggregate stability indices are presented in Figure 8. Compared with the unamended red soil, the three amendments altered the aggregate-size distribution and improved aggregate stability, although the response patterns differed among amendment types and contents. As shown in Figure 7, after LB treatments, DK- and XG-amended soils retained higher proportions of coarse aggregate fractions, whereas Ca-LS exhibited a more amendment-content-dependent variation in the retained aggregate-size distribution. After the three LB treatments, the mass percentages of water-stable aggregates > 0.2 mm in the unamended red soil, DK-amended red soil, and XG-amended red soil followed the order of WS > SW > FW, whereas the Ca-LS-amended red soil followed the order of SW > WS > FW.

Figure 7 further shows that, under the three treatment conditions, the mass percentages of aggregates > 2 mm and >0.2 mm generally increased with increasing DK and XG amendment contents. In contrast, Ca-LS exhibited a non-monotonic response, with the highest aggregate stabilization generally occurring at amendment contents of 1.0–1.5%. The variation trend of aggregate stability values under the three treatments was basically consistent with that of the aggregate-size fractions. Among them, when amendment content was 0.5%, the soil under most treatments could reach a stable or very stable state.

Overall, under fast wetting (FW), slow wetting (SW), and wet stirring (WS) treatments, the aggregates of XG-amended and DK-amended red soil both showed good stability, while the aggregates of Ca-LS-amended red soil had a high resistance to slow wetting and wet stirring treatments.

For red soil amended with 0.5–1.5% DK and 0.5% XG, the mean weight diameter (MWD) of aggregates followed the order of WS > SW > FW, consistent with that of the unamended red soil. In contrast, the MWD of aggregates in red soil amended with 2% DK, 1% XG, and all Ca-LS amendment contents exhibited the order of SW > WS > FW in turn. This suggests that the primary disintegration mechanism of aggregates in these amended red soils was slaking, followed by mechanical disturbance destruction, with differential clay swelling destruction being the weakest.

**Figure 9:**
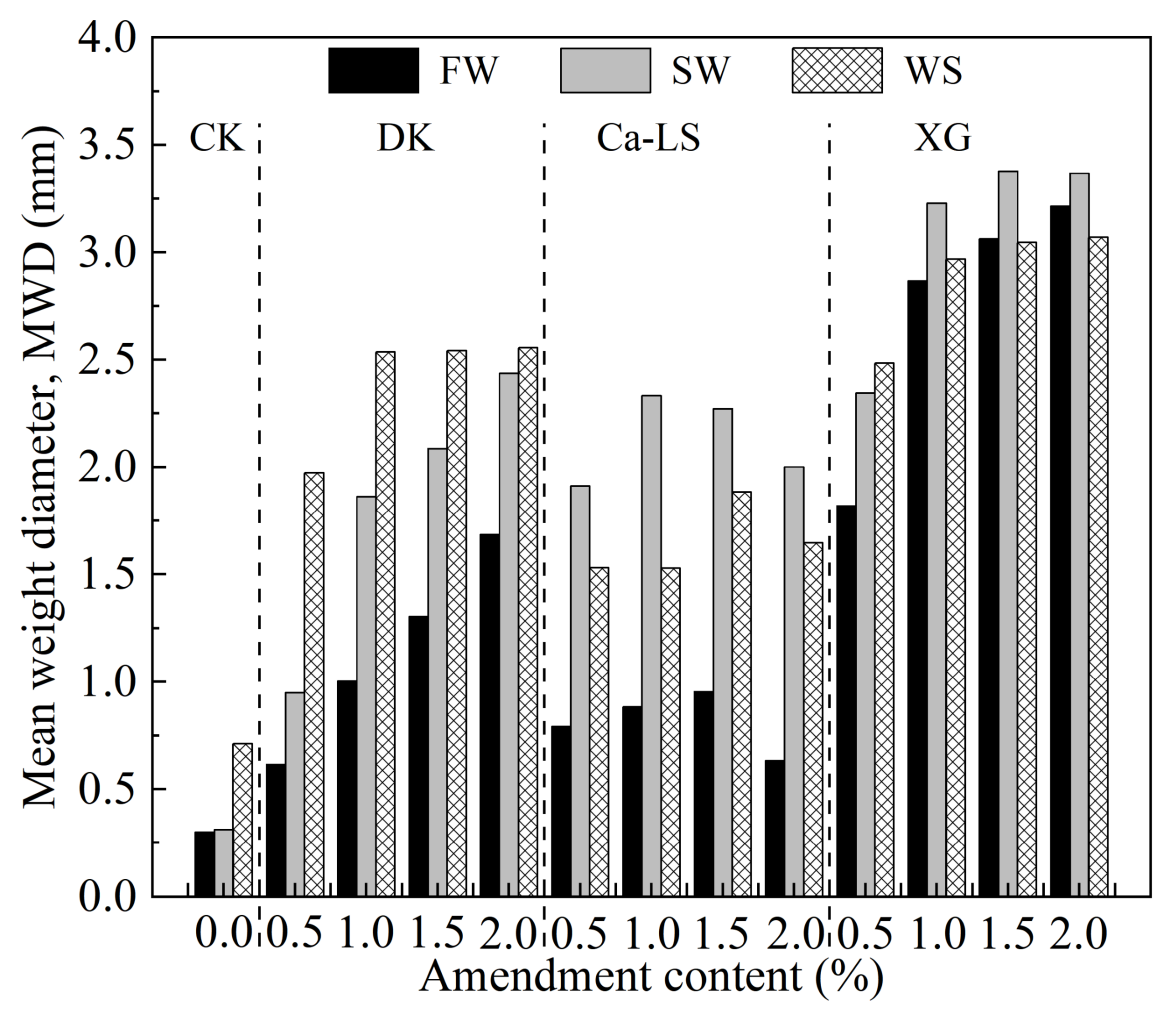
Characteristics of aggregate disintegration mechanism of amended red soil under water action.

Furthermore, the MWD of aggregates in red soil amended with 1.5–2% XG followed the order of SW > FW > WS (Figure 9). This suggests that the main disintegration mechanism of such amended red soil aggregates was mechanical disturbance destruction, followed by slaking, with minimal differential clay swelling destruction. Notably, XG at an amendment content of no less than 1.5% can alter the primary type of disintegration mechanism of red soil aggregates.

### 3.5. Zeta Potential Characteristics

**Figure 10:**
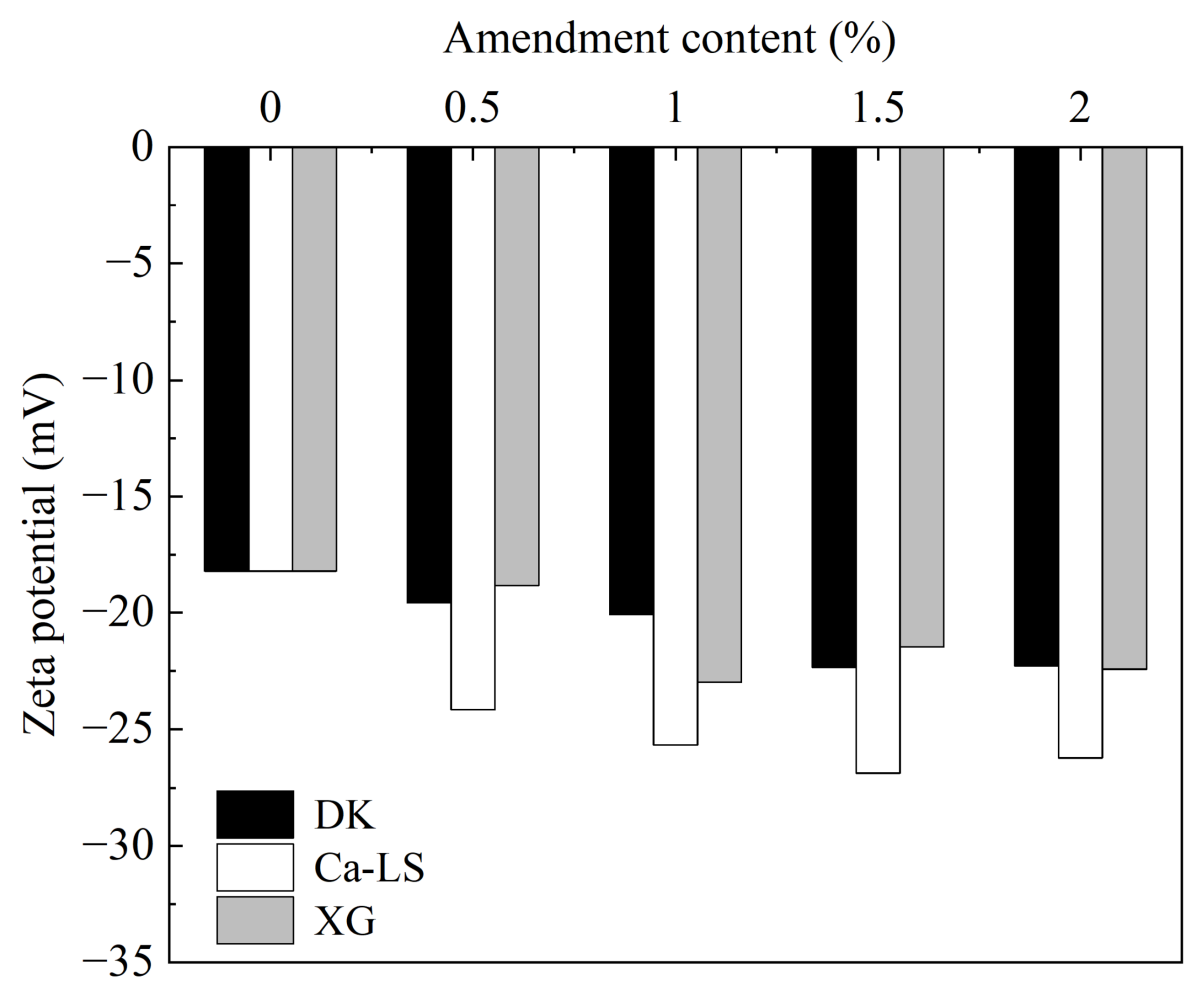
Zeta potential of unamended and amended red soil.

The zeta potentials of the unamended red soil and amended red soil are presented in Figure 10. The zeta potentials of both the red soil and its amended samples were negative, indicating that the surfaces of soil colloid particles generally carried negative charges. Under different amendment treatments, the variation trends of zeta potential differed. The zeta potential of DK-amended red soil became progressively more negative with increasing amendment content, corresponding to increases in absolute magnitude of 7.59–22.71% compared with the unamended red soil. For the XG treatment, the zeta potential showed a fluctuating trend, becoming more negative, then less negative, and then more negative again, with the most negative value observed at an amendment content of 1%, corresponding to increases in absolute magnitude of 3.43–26.23% compared with the unamended red soil. In contrast, the zeta potential of Ca-LS-amended red soil first became more negative and then less negative, reaching the most negative value at an amendment content of 1.5%, corresponding to increases in absolute magnitude of 32.72–47.63% compared with the unamended red soil.

Overall, the shifts in zeta potential induced by DK and XG were relatively mild, whereas Ca-LS caused a more pronounced shift toward more negative values. Application of the amendments generally shifted the zeta potential toward more negative values, corresponding to an increase in its absolute magnitude and suggesting enhanced electrostatic repulsion between particles. However, this electrostatic effect does not preclude amendment-mediated interparticle bonding. Zeta potential reflects the electrokinetic repulsion of particles in aqueous suspension, whereas adsorption, polymer-chain bridging and entanglement, and mineral-mediated bridging or cementation can provide attractive or adhesive interactions at particle contacts. Aggregate stability therefore reflects the balance between these competing effects rather than zeta potential alone. The observed shift toward more negative zeta-potential values was mainly ascribed to the abundant active functional groups (e.g., carboxyl and hydroxyl groups) present in the three amendments. These groups generate negative charges through hydrolysis, dissociation, or deprotonation reactions and adsorb onto the surfaces of colloid particles, thereby increasing their electronegativity.

In this study, the measured zeta potentials ranged from −26.9 to −18.2 mV, which falls within the interval where the colloid system begins to become unstable. Although the electrostatic repulsion has a limited impact on the soil structure under dry conditions, under water immersion conditions, a more negative zeta potential, and hence a greater absolute magnitude, indicates stronger electrostatic repulsion and a greater tendency toward particle dispersion. Particularly for the soil amended with Ca-LS, obvious clay loss was observed. This also explains why excessive Ca-LS addition tended to impair water stability. Therefore, although the shift toward more negative zeta-potential values may increase the tendency toward particle dispersion, amendment-induced adsorption, bridging, and cementing interactions can simultaneously strengthen interparticle bonding. The observed aggregate stability therefore reflects the net balance between electrostatic repulsion and these attractive or adhesive interactions.

## 4. Mechanisms and Discussion

### 4.1. Differential Effects of the Three Biopolymer Amendments on Aggregate Stability

This study demonstrated that XG, Ca-LS, and DK all enhanced the stability of red soil aggregates under fast wetting (FW), slow wetting (SW), and wet stirring (WS) conditions, although their effectiveness, optimal amendment content ranges, and response patterns differed substantially. The results indicate that aggregate stabilization in red soil is not governed by amendment type alone, but rather by the interaction between amendment chemistry and the intrinsic physicochemical properties of the soil. These findings are generally consistent with previous studies showing that biopolymer-based amendments can improve soil aggregation and resistance to water-induced disintegration [30].

XG exhibited a pronounced stabilizing effect at a relatively low amendment content, approximately 1%. This was evidenced by the rightward shift in aggregate-size distribution, the increases in mean weight diameter (MWD) and water-stable aggregates (WSAs), and the formation of a dense and continuous cementing structure observed by SEM. This behavior is consistent with the typical stabilization mechanisms of polysaccharide biopolymers, which rapidly strengthen interparticle bonding through molecular chain entanglement, viscous cementation, and hydrogen bonding [31,32]. The amended-soil FTIR spectra further showed that the band near 1032 cm^−1^ shifted to approximately 1028 and 1026 cm^−1^ at 1% and 2% XG, respectively. This fingerprint-region shift is consistent with modification of the local mineral–polymer interfacial environment after XG addition. In contrast, Ca-LS showed a more distinct “window-type” amendment content response, whereby an appropriate amendment content improved aggregate stability, whereas excessive addition reduced the stabilizing effect (Figure 9). This pattern was consistent with the changes in Zeta potential and SEM morphology. The FTIR spectra further showed a blue shift in the broad O-H stretching band from approximately 3421 cm^−1^ in the unamended red soil to 3442 and 3437 cm^−1^ at 1% and 2% Ca-LS, respectively. This spectral shift indicates a reorganization of the local O-H bonding environment and is consistent with amendment–soil interfacial interactions. At suitable amendment contents, Ca-LS may promote particle flocculation and bridging through Ca^2+^ bridging, charge shielding, and adsorption of lignosulfonate molecular chains [33]. These effects are not inconsistent with the more negative zeta potential, because electrostatic repulsion in the suspension can coexist with adsorption- and Ca^2+^ -mediated interparticle bridging at particle contacts. However, at excessive amendment contents, the accumulation of anionic groups may enhance electrostatic repulsion among particles, leading to fine-particle dispersion and structural deterioration. Similar amendment content-dependent and electrostatically regulated effects have also been reported in studies on lignosulfonate and other anionic surface-active materials used for soil stabilization [34,35,36].

Unlike XG, which produced a strong stabilizing effect at low amendment content, and Ca-LS, which showed a relatively narrow effective amendment content range, DK exhibited a gradual increase in aggregate-stabilizing effectiveness with increasing amendment content. This response may be related to its organic–mineral composite composition. The improvement in aggregate stability increased progressively with DK amendment content and reached a relatively high level at higher amendment contents. SEM observations showed that DK amendment generated fine cementing materials that were relatively uniformly distributed on particle surfaces and at interparticle contact points, rather than forming a continuous network structure as observed for XG or coarse bridging structures as observed for Ca-LS. Nitrogen adsorption results further showed that DK reduced the specific surface area of red soil from 34.42 to 30.94 m^2^ g^−1^ and increased the average pore diameter from 7.91 to 9.26 nm (Figure 5). These results suggest that DK gradually enhanced aggregate stability by coating fine-particle surfaces, promoting the aggregation of micro-sized particles, and modifying the pore structure.

At the interfacial scale, the hydroxyl- and mineral-related bands identified in DK, together with the shift in the broad band near 3421 cm^−1^ in unamended red soil to approximately 3446 cm^−1^ after 2% DK amendment, indicate an alteration of the local interfacial bonding environment and are consistent with surface adsorption of DK onto soil particles. Meanwhile, its inorganic mineral component may act mainly as a fine-particle filler and structural framework, while the mineral surfaces may provide adsorption sites for the organic components, thereby promoting organic–mineral interfacial coupling rather than ionic bridging. The zeta potential results showed that DK induced changes in zeta potential ranging from 7.59% to 22.71%, which were weaker than those induced by Ca-LS. This suggests that DK does not primarily rely on strong electrostatic regulation, but more likely enhances interparticle bonding progressively through the combined effects of mineral bridging and organic interfacial adhesion.

Therefore, the stabilization mechanism of DK should not be simply interpreted as biopolymer cementation. Instead, it should be understood as an organic–mineral coupling-dominated particle-scale stabilization process. Its organic components may contribute to hydrogen bonding, surface adsorption, and interfacial adhesion, whereas its mineral components may contribute to fine-particle filling, structural support, and the provision of adsorption sites for organic coatings. These effects may jointly promote interparticle bonding and pore-structure reorganization, which may help explain the gradual enhancement of DK-induced stabilization with increasing amendment content. It should be noted that, because no pure mineral control was included in this study, the relative contributions of the mineral and organic components in DK could not be quantitatively distinguished. Therefore, the present study mainly provides qualitative and descriptive evidence, and quantitative assessment of the respective roles of different DK components should be a priority in future research. Taken together, the dominant stabilization pathways inferred from the present results differ among the three amendments. The stabilization behavior of XG was closely associated with its polymeric network formation. In contrast, the response of Ca-LS appeared to depend more strongly on adsorption-related interactions and Ca^2+^ involvement. For DK, the observed changes are better explained by the combined effects of its organic and mineral components.

### 4.2. Multiscale Mechanisms Underlying Aggregate Stabilization

Overall, the effects of the amendments on aggregate stability should not be interpreted as a single chemical cementation process, but rather as the combined outcome of enhanced interfacial bonding, microstructural reorganization, and adjustment of the pore system.

At the interfacial scale, the treatment-dependent FTIR spectral changes were consistent with alterations in the local O-H and mineral–organic interfacial bonding environments, while the zeta-potential results suggested additional contributions from ionic interactions and charge regulation. For highly weathered red soil, the high fine-particle content, large specific surface area, and strong activity of Fe and Al oxides provide abundant reactive sites for biopolymer adsorption and mineral–organic association. Therefore, although the three amendments followed different dominant stabilization pathways, their common mechanistic basis was the strengthening of interparticle connections, thereby reducing the likelihood of particle dispersion and detachment under water-induced disturbance. This interpretation is also consistent with previous studies suggesting that the effectiveness of soil amendments is jointly controlled by biopolymer molecular structure, surface charge characteristics, and soil mineralogical background [37].

At the microscale, amendment addition changed the morphology of interparticle connections. SEM observations showed that the amended treatments generally enhanced cementation and bridging at particle contact points, transforming the originally loose particle structure with clear boundaries into a more compact composite structure (Figure 4). Soil structural function is not determined solely by aggregate size, but also by microstructural characteristics such as particle arrangement, pore geometry, and connectivity. These characteristics influence water entry, stress transmission, and particle detachment processes. Schlüter et al. [9] emphasized that pore-scale imaging can reveal the close relationship between soil structure and soil function, particularly the importance of pore morphology and connectivity for water and gas transport as well as structural stability. Menon et al. [38] further showed that the internal pore system of aggregates is closely related to aggregate stability, and that changes in pore-size distribution and connectivity can affect the resistance of aggregates to water-induced breakdown. In the present study, the enhanced cementation and bridging observed by SEM corresponded well with the increases in MWD and the proportion of large water-stable aggregates, indicating that microstructural reorganization provides an important structural basis for the improved stability of red soil aggregates.

At the pore scale, the amendments induced adjustments in the internal pore structure of aggregates. All three amendments altered the BJH-resolved mesopore distribution and generally increased the proportion of pores within the 10–50 nm range, suggesting the simultaneous occurrence of fine-particle aggregation and pore rearrangement (Figure 6). Similar pore-structure optimization has also been observed in soils amended with biopolymer- or carbon-based amendments, where improved pore structure is generally associated with more favorable water–air distribution and enhanced structural stability [39,40]. Under fast wetting conditions, aggregate breakdown is commonly associated with rapid water entry, compression of entrapped air within pores, and the resulting increase in local pore pressure [41]. Therefore, the transformation of pores from finer to relatively larger pore sizes may facilitate water entry, alleviate internal stress concentration caused by rapid wetting, and consequently enhance aggregate resistance to slaking [42]. The study by Roosch et al. [43] on the contrasting mechanisms of water stability in macroaggregates and microaggregates also indicates that mechanical stability and water stability are not necessarily equivalent, but are closely related to internal aggregate structure, pore characteristics, and failure processes.

The improvement in red soil aggregate stability should therefore be understood as a multiscale coupled process, involving enhanced interfacial interactions that strengthen interparticle bonding, microstructural reorganization that improves aggregate integrity, and pore-structure adjustment that modifies water entry and disturbance response. The differences among the three amendments lie in the relative dominance of these processes at different scales [37]. XG mainly promoted interfacial adhesion and network reinforcement, Ca-LS was more closely associated with charge regulation and particle bridging, and DK primarily relied on coupled organic–inorganic mineral interfacial bonding and mineral filling. These scale-dependent observations explain why the three amendments exhibited different responses under FW, SW, and WS treatments.

### 4.3. Environmental Implications, Application Boundaries, and Study Limitations

The results of this study indicate that the improvement of red soil aggregate stability depends not only on the intrinsic stabilizing capacity of the amendments, but also on the degree of matching between their stabilization pathways and the dominant breakdown mechanisms. Therefore, in practical applications, amendment selection should not be based solely on a single stability index, but should also consider soil properties, rainfall disturbance patterns, and the stage of restoration in the target area [40]. For preliminary laboratory screening, XG showed a marked aggregate-stability response at comparatively low product contents, Ca-LS showed a narrower favorable content range, and DK exhibited gradual improvement with increasing content. Their suitability for engineering applications remains to be validated under field rainfall, durability, cost, and ecological-safety constraints.

This study also has several limitations. First, the experiments were conducted using only one type of red soil under laboratory conditions; therefore, the applicability of the results to different soil types, rainfall regimes, and field environments requires further validation. Second, although SEM, FTIR, pore-structure characterization, and zeta potential measurements were integrated to interpret the stabilization mechanisms, the relative contributions of specific processes, such as hydrogen bonding and mineral structural contributions, were not quantitatively distinguished. In addition, because DK is a composite amendment and no pure mineral control was included in this study, the synergistic contributions of its mineral and organic components remain to be further clarified.

Overall, this study suggests that the DK organic–mineral composite amendment may combine the interfacial adhesion advantages of organic materials with the filling, structural-support, and surface-interaction functions of inorganic components, thereby providing a new strategy for the structural restoration of degraded red soil that differs from single-biopolymer amendment approaches. Future research should further focus on optimizing component ratios, evaluating long-term stability, assessing ecological safety, and verifying field applicability, so as to promote the use of such composite amendments in soil restoration and ecological engineering.

## 5. Conclusions

This study systematically evaluated the effects of xanthan gum (XG), calcium lignosulfonate (Ca-LS), and a DK bio-organic–mineral composite stabilizer on the stability of red soil aggregates, and further explored their stabilization mechanisms by integrating microstructural observations, pore characteristics, FTIR spectra, and zeta potential analyses. The main conclusions are as follows:

(1) All three amendments improved the stability of red soil aggregates, but they exhibited distinct amendment content patterns. XG markedly enhanced aggregate stability even at a relatively low amendment content, Ca-LS showed a strong stabilizing effect within an appropriate amendment content range but had a relatively narrow effective amendment content window, and DK exhibited a gradual increase in stabilization effectiveness with increasing amendment content. Within the amendment content range tested in this study, the optimal amendment contents of XG, Ca-LS, and DK were 1.0%, 1.5%, and 2.0%, respectively.
(2) The stabilizing effects of the three amendments corresponded closely to their microstructural and interfacial interaction characteristics. XG formed a relatively continuous cementing network even at low amendment content, indicating its capacity to rapidly enhance aggregate stability. Ca-LS promoted strong particle bridging at an appropriate amendment content, whereas excessive addition may increase the risk of fine-particle dispersion due to overly strong electrostatic regulation. DK formed fine and uniformly distributed particle-scale cementing structures, accompanied by a decrease in specific surface area and an increase in average pore diameter. These results suggest that its stabilizing effect may originate from an organic-mineral coupling mechanism involving organic interfacial adhesion, mineral filling, and structural support.
(3) The improvement in red soil aggregate stability essentially resulted from the coupled effects of enhanced interfacial bonding, structural reorganization, and pore regulation. Different amendments participated in this process through different dominant pathways, thereby leading to their contrasting stabilizing effects under hydraulic and mechanical disturbance conditions.

From an engineering perspective, amendment selection should account for site-specific environmental conditions, material properties, and dosage, with practical applicability further validated through field assessments of durability, cost-effectiveness, and ecological safety.

## Figures and Tables

**Table 1:** Experimental Design for the Preparation and Treatment of Red Soil Aggregate Specimens.

Soil Amendments	Amendment Content/%	Initial Water Content/%
DK	0, 0.5, 1, 1.5, 2	18
Ca-LS	0, 0.5, 1, 1.5, 2	18
XG	0, 0.5, 1, 1.5, 2	18

Note: The amendment content is defined as the mass ratio of the commercial amendment product to oven-dried soil.

**Table 2:** Experimental Design for the Water Stability Improvement Mechanism of Red Soil.

Test Items	Amendment Contents/%		
	DK	Ca-LS	XG
Scanning Electron Microscopy (SEM) Test	0, 1.5	0, 1.5	0, 1.5
Nitrogen Adsorption–Desorption Test	0, 0.5, 1, 1.5, 2	0, 0.5, 1, 1.5, 2	0, 0.5, 1, 1.5, 2
Zeta Potential Analysis	0, 0.5, 1, 1.5, 2	0, 0.5, 1, 1.5, 2	0, 0.5, 1, 1.5, 2
Fourier Transform Infrared (FTIR) Spectroscopy Test	0, 1, 2	0, 1, 2	0, 1, 2

## Data Availability

The original contributions presented in this study are included in the article. Further inquiries can be directed to the corresponding author.
